# Rubella infection in pregnancy and congenital rubella in United Kingdom, 2003 to 2016

**DOI:** 10.2807/1560-7917.ES.2018.23.19.17-00381

**Published:** 2018-05-10

**Authors:** Antoaneta Bukasa, Helen Campbell, Kevin Brown, Helen Bedford, Mary Ramsay, Gayatri Amirthalingam, Pat Tookey

**Affiliations:** 1 Public Health England, Immunisation- Hepatitis and Blood Safety, London, United Kingdom; 2 Public Health England, Virology Reference Department, London, United Kingdom; 3 UCL Great Ormond Street Institute of Child Health, Faculty of Population Health Sciences Population, London, United Kingdom

**Keywords:** rubella, congenital rubella syndrome, pregnancy, MMR vaccine, epidemiology, rubella elimination

## Abstract

Rubella vaccination has been included in the United Kingdom’s (UK) routine childhood schedule for nearly 30 years. The UK achieved World Health Organization (WHO) elimination status in 2016 and acute rubella infections are rare. In the period 2003–16, 31 rubella infections in pregnancy (0.23 per 100,000 pregnancies) were identified through routine surveillance, of which 26 were in women who were born abroad. Five of the 31 rubella infections led to congenital rubella syndrome in the infant and three had confirmed congenital rubella infection without congenital rubella syndrome. An additional seven babies were identified with congenital rubella syndrome, although rubella infection in pregnancy had not been reported. Place of birth was known for six of these seven mothers, all of whom were born outside the UK, and in five cases maternal infection was acquired abroad. WHO Europe has set targets for measles and rubella elimination and prevention of congenital rubella syndrome by 2015. Vaccination uptake and rubella immunity is high in the UK population and most infections in pregnancy since 2003 were acquired abroad and in unvaccinated women. Every contact with a health professional should be used to check that women are fully immunised according to UK schedule.

## Introduction

Although rubella is usually a mild, sometimes asymptomatic illness in childhood, the consequences of rubella infection in pregnancy can be devastating. In 2010, the Pan American Health Organisation announced that the Region of the Americas had eliminated rubella and congenital rubella syndrome (CRS) [1,2]. In the same year all 53 Member States of the World Health Organization (WHO) European Region committed to the goal of eliminating endemic transmission of measles and rubella, initially by 2015 and later revised to 2020. For the elimination of rubella and congenital rubella, high coverage of a two-dose childhood vaccination programme of a rubella-containing vaccine must be sustained [3]. Rubella can be easily mistaken for a number of other viral infections, and in order to monitor progress towards elimination it is essential that countries across Europe have robust surveillance systems in place to identify all suspected cases and reliably confirm or exclude rubella and congenital rubella infection (CRI) using appropriate laboratory methods [4].

Surveillance systems and laboratory confirmation of rubella and congenital rubella cases vary across Europe. Despite the elimination goals set for the WHO European Region, three of 28 European Union (EU) countries do not have national surveillance systems for all rubella cases [5,6]. In 2016, only 5% of all cases reported to the European Centre for Disease Prevention and Control (ECDC) were laboratory-confirmed. The United Kingdom (UK) vaccination strategy and programme surveillance is very similar to other western European countries and is based on laboratory-confirmed cases. It is, however, the only country to perform routine IgM confirmatory testing of oral fluid of notified cases (since 1994), which has strengthened surveillance and improved ascertainment [7-9].

The consequences of rubella infection in the first 20 weeks of pregnancy, and the relationship between gestational week of exposure and likelihood of fetal loss or features of congenital rubella syndrome, have been well documented [10,11]. With the introduction of effective vaccination strategies in the UK, the incidence of rubella has decreased dramatically and the last large outbreak of rubella occurred in 1995–96. Most clinicians who have qualified in this country in the past 20 years will never have seen a case of rubella, rubella infection in pregnancy or congenital rubella.

Before routine vaccination was introduced, rubella was a common childhood disease in the UK with 80% of adults having evidence of prior infection [12]. Rubella vaccination was introduced for susceptible women and girls aged 11–14 years in 1970 with the aim of allowing most girls to acquire natural immunity in earlier childhood [13,14]. Non-immune women of child-bearing age were also targeted following the introduction of antenatal screening for rubella susceptibility based on rubella IgG testing throughout the UK in the early 1970s. The main programme aim was to ensure women of childbearing age were immune to rubella and thus prevent primary infection in pregnancy. This strategy successfully increased the proportion of women with antibodies to rubella from 85–90% in 1970 to 97–98% in 1987 [15]. Surveillance of CRS and CRI infections was established in the UK in 1971 to monitor the effectiveness of the vaccination programme [16].

While programmes to directly protect women of childbearing age against rubella successfully reduced cases of congenital rubella and terminations following rubella infection in pregnancy [16,17], the disease continued to circulate among young children, who were a potential source of infection to any women who remained susceptible. In 1988, a combined measles-mumps-rubella (MMR) vaccination was introduced into the routine childhood schedule at 12–15 months of age. The rubella component of MMR vaccine is highly effective and a single dose of a rubella-containing vaccine confers around 95–100% protection; the measles and mumps components require two doses to reach high levels of effectiveness [10,18]. A successful mass school-based measles-rubella immunisation campaign targeting all children aged 5–16 years was conducted in 1994 to prevent a predicted measles epidemic and to address continuing high levels of rubella susceptibility in school-aged children, particularly among boys [15]. To ensure continued high population protection, a routine second dose of MMR for 3–4-year-olds was introduced from 1996, when selective immunisation of schoolgirls ended. Uptake of the two-dose schedule by fifth birthday in the UK reached 75% by June 2005 [19] and was 88.5% in October to December 2016 [20]. MMR first-dose coverage by fifth birthday reached the 95% WHO elimination target for the first time in 2016.

Determination of rubella susceptibility is not straightforward. The widespread use of an ELISA cut-off value of 10IU/ml, generally accepted as evidence of immunity, is based on levels following vaccination [21]. Vaccine-induced rubella antibody levels, while protective, appear to be lower at a population level than those resulting from naturally acquired infection. With an increasing proportion of UK-born women acquiring immunity through vaccination rather than natural infection and the absence of circulating rubella in the UK, reported antenatal susceptibility rates have increased in recent years based on this cut-off value [9].

In 2013, 27% of all births in England were to women born outside the UK, with geographical variation peaking in London at 58% [22]. Many of these women are likely to have come from rubella-endemic countries, with no or disrupted routine immunisation against rubella. A higher proportion of non-UK-born mothers, particularly those from sub-Saharan Africa and south-east Asia were more likely to be seronegative than UK-born women or white British women [23], [24] and analysis of antenatal rubella susceptibility data from London suggested that between 16% and 65% of non-UK-born women were susceptible in 2007 [25].

This paper summarises cases of laboratory-confirmed rubella infection in pregnancy (IIP), CRI and CRS reported to Public Health England (PHE) and other national surveillance programmes in the UK between 2003 and 2016.

## Methods

### National rubella enhanced surveillance scheme

Rubella has been a notifiable disease in the UK since 1988, with health professionals legally required to report all clinically diagnosed cases. Since 1995, oral fluid samples have been requested for all notified rubella cases in order to confirm or refute the clinical diagnosis [9] regardless of local testing. Confirmed rubella cases are followed up by an enhanced surveillance form, completed by the general practitioner or hospital doctor, to ascertain further details, including vaccination history, demographics, contacts and travel outside the UK in the month preceding onset of symptoms. Details of pregnancy outcome are sought from those responsible for the care of the woman and, in the case of a live birth, for her infant along with details of clinical presentation and samples for further laboratory analysis. Where possible, retrospective laboratory investigations of maternal pregnancy samples are carried out when infants are diagnosed with CRS or CRI after birth but when the infection is not diagnosed in the mother during pregnancy.

### National Congenital Rubella Surveillance Programme (NCRSP)

Established in 1971 at the Institute of Child Health (London), the National Congenital Rubella Surveillance Programme (NCRSP) seeks reports of all suspected and confirmed cases of congenital rubella from paediatric respondents to the Royal College of Paediatrics and Child Health’s British Paediatric Surveillance Unit in the UK and Republic of Ireland [26].

### Hospital Episodes Statistics

Patients were identified using the Hospital Episode Statistics (HES) database which contains details of all admissions to National Health Service hospitals in England. Admissions between 1 April 2002 and 31 March 2017 with an ICD-10 code for congenital rubella syndrome (P35.0) or maternal care for (suspected) damage to fetus from viral disease in mother (O35.3) in the primary diagnosis field were selected. A re-admission within a 180-day period was treated as the same episode.

### Laboratory testing of suspected rubella infection in pregnancy and congenital rubella syndrome

For all suspected rubella cases in pregnant women, paired serum samples are requested in order to confirm the diagnosis and distinguish between primary rubella infection and reinfection. Primary rubella infection is confirmed by a combination of rubella IgM plus rubella IgG seroconversion, detection of rubella virus RNA and/or detection of low-avidity rubella antibody. A diagnosis of rubella reinfection (including infection in someone who has previously been vaccinated) is made if there is a significant increase in rubella IgG and the rubella IgG is of high avidity.

Samples of cord blood, placenta, urine and an oral fluid sample are taken from the infant soon after delivery. CRI is confirmed by detection of rubella IgM in serum or oral fluid and/or detection of rubella RNA in body fluids, and those infants who also have clinical features consistent with congenital rubella syndrome are classified as CRS cases [4].

## Results

### Rubella infections in pregnancy

**Figure 1:**
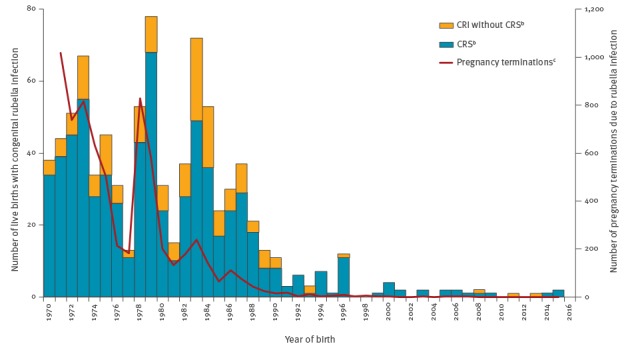
Infants with congenital rubella infection and those diagnosed with congenital rubella syndrome^a^ (live births) and pregnancy terminations due to rubella infection during pregnancy, United Kingdom^b^, 1970−2016

NCRSP: National Congenital Rubella Surveillance Programme; UK: United Kingdom.

^a^ Congenital rubella infectionis confirmed by detection of rubella IgM in serum or oral fluid and/or detection of rubella RNA in body fluids, who also have clinical features consistent with congenital rubella syndrome are classified as CRS cases.

^b^ The NCRSP scheme was initially established to monitor pregnancies in England and Wales. Scottish data was added subsequently to the historical data for England and Wales. Information on Northern Ireland data is not available before 1991.

^c^ Disease or contact, England and Wales only. Data on terminations from Scotland (7% of UK live births) and Northern Ireland (3% of UK live births) are not available.

Source: National Congenital Rubella Surveillance Programme and for rubella-associated terminations: Department of Health and Office for National Statistics.

Over this 14-year period, 31 of 270 confirmed rubella infections were in pregnant women. There was therefore an average annual incidence of 0.23 rubella infections per 100,000 pregnancies (95% confidence interval (CI): 0.16–0.33/100,000). Over the time period covered in this report, there were an average 24 rubella cases, 2.6 IIPs and 1.3 CRS births reported annually between 2003 and 2009 and an annual 15 rubella cases, 1.9 IIPs and 0.4 CRS births on average between 2010 and 2016. This continues a substantial decline observed over the past 30 years (Figure 1). An average of just over 20 CRS births a year were reported between 1986 and 1990, with 3.3 on average each year between 1991 and 2002 in Great Britain [17].

### Rubella reinfection in pregnancy

**Figure 2:**
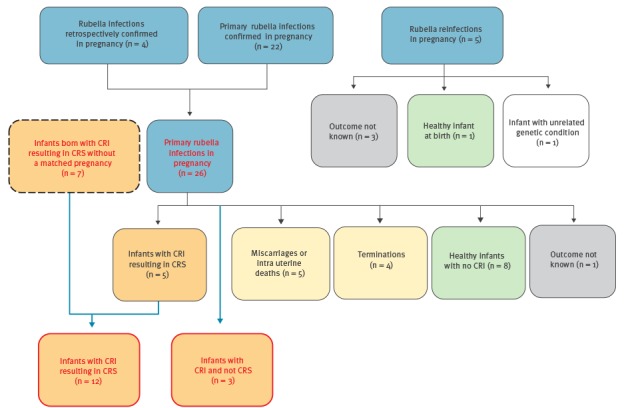
Details of confirmed rubella exposures in pregnant women (n = 31) and congenital rubella infections identified after delivery (n = 7), United Kingdom, 2003–2016

CRI: congenital rubella infection; CRS: congenital rubella syndrome.

Of the 31 infections identified in pregnancy, five were classified as reinfection rather than primary infection (Figure 2). The mean age of these five women was 32 years. In one of these reinfections the pregnancy led to a healthy infant, in three the outcome was not known and in the fifth the infant had trisomy 21 and no further rubella testing was undertaken.

### Primary rubella infection in pregnancy

Twenty-six of the 31 infections during pregnancies were due to primary infection. In four women, infection was confirmed retrospectively using stored samples following investigation of their infant after delivery (Figure 2). The mean age of the 26 women at diagnosis was 27 years (range 16 – 41 years). Country of birth was reported for 20 of the 26 women, all of whom were non-UK-born (Table 1): eight were born in four other European countries; five in Africa; five in Asia; two were born in the Americas. Among the 22 primary infections with relevant information available, 14 women were reported to have acquired their infection outside the UK, either before entering the UK for the first time or while travelling abroad. Of the remaining eight women, six were known to have been born abroad and were known to or were likely to mix socially with other non-UK born populations. In line with seroprevalence data, geographically the majority of women presented in London (14/26, Table 1) with the rest distributed throughout the UK. Only one of these women reported prior immunisation with rubella-containing vaccine but her laboratory results were consistent with primary infection.

**Table 1:** Summary of reported primary rubella infections in pregnancy with outcomes, United Kingdom, 2003–2016 (n = 26^a^)

**Confirmed rubella infection in pregnancy**		
Primary infection confirmed in pregnancy		22
Reported retrospectively (infection in pregnancy confirmed post-delivery)		4
**Susceptibility and acquisition**		
Place of birth	Woman born abroad	20
	Woman born in the UK	0
	Details not available on place of birth	6
Place of infection acquisition	Maternal infection acquired abroad	14
	Maternal infection acquired in the UK	8
	Details not available on place of infection acquisition	4
Place of residence	Residing in London	14
	Residing outside London	12
**Pregnancy outcomes**		
Intrauterine death / stillbirth		3
Miscarriage		2
Termination (due to IIP)		4 (3)
Healthy infant		8
Infant with CRI but no CRS features at birth		3
Infant with CRI resulting in CRS		5
Not known		1

CRI: congenital rubella infection; CRS: congenital rubella syndrome; IIP: infection in pregnancy; UK: United Kingdom.

^a^ Excluding five reinfections in pregnancy and seven congenital rubella infection cases identified post-delivery with no matched maternal record.

### Outcomes following primary infections

Of the 26 pregnancies; four ended in termination (three reported to be directly due to the rubella infection) and five resulted in miscarriage or intrauterine death (IUD)/stillbirth (Table 1). Samples were not available in these nine cases without a live birth but it was known that one termination and one IUD were considered to be unrelated to the rubella IIP. Eleven pregnancies resulted in babies who were asymptomatic at birth, eight of whom were free of infection and three had confirmed CRI without CRS. Five babies had CRI with CRS. The outcome for one of the 26 pregnancies was not known.

### Congenital rubella syndrome in infants without prior confirmation of maternal infection

There were seven babies with confirmed CRI reported through the enhanced surveillance conducted by the NCRSP and laboratory investigation of babies undertaken by the PHE (up to 2013, by the Health Protection Agency (HPA)) Virus Reference Department without laboratory confirmation of maternal infection during pregnancy (Table 2). All seven babies had features of CRS. Place of birth was known for six of the seven mothers, all of whom were born outside the UK. In five cases, maternal infection was acquired abroad: four mothers were recent arrivals from Indian subcontinent or Africa; one mother acquired her infection while travelling in Europe. In addition, one mother had not travelled but was part of community with known links to the Indian subcontinent and infection in one mother was missed as she did not present with symptoms in pregnancy.

**Table 2:** Summary of congenital rubella infections, United Kingdom, 2003–2016 (n = 15^a^)

	Features or characteristics	Total
Diagnosed with infection in pregnancy	Infant with CRI but no CRS features at birth	3
	Infant with CRI resulting in CRS	5
Diagnosed after birth	Infant with CRI resulting in CRS	7
Acquisition of rubella infection	Maternal infection acquired abroad	8
	Maternal infection UK acquired	6
	Place of infection not known	1
Maternal place of birth	Mother born abroad	14
	Maternal place of birth not known	1
Reported features of 11 of 12 CRS cases^b^ (more than one feature may be reported for each infant)	Thrombocytopaenia at birth	2
	Sensorineural hearing loss	10
	Eye defects	8
	Failure to thrive	3
	Heart defects	10
	Developmental problems	3
	Microcephaly	5

CRI: congenital rubella infection; CRS: congenital rubella syndrome; UK: United Kingdom.

^a^ Including seven babies with congenital rubella infections identified post-delivery with no matching maternal record.

^b^ More than one feature may be reported for each infant. One infant was reported as a CRS case but further details were not available and this infant is therefore excluded from the summary of reported features.

Analysis of the HES database revealed two additional patients with CRS codes that were unknown to both the NCRSP or through the enhanced rubella surveillance scheme. When further information was obtained, both cases were discarded as they did not fit the criteria for inclusion; one was born outside the UK and the other had an alternative diagnosis.

### Features of cases of congenital rubella syndrome in the United Kingdom

In nine of the 12 cases of CRS (combining those identified through confirmed rubella IIP and post-delivery,) gestational age at infection was known and in each case occurred in the first 16 weeks of pregnancy. Eight healthy babies without infection were delivered following rubella exposure after 16 weeks. Two infants with CRS died in their first year, although in one case this was considered unrelated to CRS. Over the 14-year period, reported CRS rates averaged 0.1 per 100,000 (95% CI: 0.06–0.20/100,000) live births annually between 2003 and 2016.

## Discussion

Twenty-four of the 53 countries in the WHO European Region, (16 of which are within the EU/European Economic Area (EEA)) achieved the elimination goal for rubella (based on 2015 data) and 11 countries, six of them in the EU/EEA, were considered to have interrupted endemic rubella transmission for less than 36 months and so are on target to achieve the elimination goal [5]. Significant progress has therefore been made in Europe and robust systems for the notification and investigation of suspected cases together with effective vaccination programmes underpin this progress. However, outbreaks still continue to occur, with Poland and Romania, for example, accounting for over 80% of reported rubella cases in Europe since 2010 [27]. Oral fluid testing of notified cases in the UK has been key to delivering a surveillance system based on laboratory-confirmed cases of rubella in line with WHO guidelines. Rubella is often a mild disease and it can be difficult to provide motivation for more invasive testing but no other country has adopted this approach.

MMR coverage has been increasing in the UK in recent years, achieving an overall 95.4% and 88.0% uptake in the last quarter of 2016 for the first and second dose respectively by the fifth birthday, although this still remains below the WHO EURO target [20]. The introduction of rubella immunisation for all children nearly 30 years ago and the subsequent change to a two-dose MMR vaccination programme in 1996 has, however, reduced rubella and rubella-linked terminations dramatically (Figure 1).

As observed in earlier years, most of the recent cases of CRS in the UK have been infants whose mothers were born abroad. Women born overseas are most at risk being left unprotected where there is a lack of rubella vaccination programmes in their country of origin or sub-optimal coverage, and the potential for missing out on catch-up after entry to the UK. They are also most at risk of being exposed to rubella through continued contact with friends and relatives living in or visiting from endemic countries. Gavi, the Vaccine Alliance has identified 57 countries previously not undertaking routine rubella vaccination and in which rubella epidemics have been reported, in which it will back the introduction of rubella vaccination to support the global eradication of rubella [28].

A number of these women were recent entrants to the UK, including some who acquired rubella infection in their country of origin. In a few cases however, women had been living in the UK for a number of years following arrival in childhood but after the age of routine MMR vaccination. Despite recommendations that the vaccination status of individuals arriving in the UK should be assessed at every possible opportunity, including school health checks, some of the recent CRS cases have highlighted potential missed opportunities for ensuring women were fully protected before pregnancy.

In fact, only one woman who acquired rubella infection in pregnancy reported a history of vaccination. In addition, while eight of 22 women had not travelled abroad, detailed follow-up revealed that they had acquired their infection from other non-UK born individuals who had recently acquired infection in an endemic country. This underlines the importance of ensuring that everyone who registers with a general practitioner has their immunisation history checked to ensure they are fully immunised according to the UK recommended vaccination schedule. It is also important to check immunisation history when children present for their routine vaccinations at school entry, transition to secondary school, teenage immunisation sessions, university immunisation sessions or opportunistically in general practice.

The antenatal screening programme was not designed to identify infection in pregnancy, but to protect subsequent pregnancies by identifying women requiring post-partum vaccination. On the recommendation of the UK Screening Committee, screening for rubella susceptibility in pregnancy was discontinued in 2016 across the UK [29] This was in the context of consistently low levels of disease in the UK, few cases of rubella infection in pregnancy and congenital rubella (meeting the World Health Organization definition of ‘elimination’) [3] and improved childhood vaccination coverage. The current guidance is that general practitioners should continue to offer MMR to non-pregnant women of childbearing age who are unvaccinated or have received one dose of a rubella-containing vaccine. Postnatally, women’s immunisation status should be checked during health visitor reviews and at 6-week maternal checks. Women accessing pre-conceptual, fertility or miscarriage and termination services should also be assessed and immunised when required.

## Conclusion

Despite the success of the vaccination programme in the UK, rubella has not been entirely eliminated and, of five CRS cases documented in the past 7 years, two could have been prevented through appropriate immunisation in the UK. Recent cases have also highlighted the importance of investigating rash illness in pregnancy according to national guidance and correct interpretation of laboratory results [30]. Further efforts should focus on ensuring that, in particular, individuals who are born overseas are offered every opportunity to be fully immunised according to the UK immunisation schedule to ensure women are fully protected before pregnancy. These issues are also likely to be key in other European countries that have achieved or are on target to achieve the elimination goal for rubella.
